# Bis(2,9-dimethyl-1,10-phenanthroline-κ^2^ *N*,*N*′)bis­(nitrato-κ^2^ *O*,*O*′)lead(II)

**DOI:** 10.1107/S1600536810022312

**Published:** 2010-06-16

**Authors:** Fwu Ming Shen, Shie Fu Lush

**Affiliations:** a Department of Biotechnology, Yuanpei University, HsinChu, Taiwan 30015; b Department of Medical Laboratory Science Biotechnology, Yuanpei University, HsinChu, Taiwan 30015

## Abstract

In the title complex, [Pb(NO_3_)_2_(C_14_H_12_N_2_)_2_], the lead ion is chelated by two 2,9-dimethyl-1,10-phenanthroline (dmphen) ligands and two nitrate anions in a slightly distorted square-anti­prismatic geometry. Intra- and inter­molecular π–π stacking is present in the crystal structure, and the centroid–centroid distances between the benzene and pyridine rings of adjacent dmphen ligands are 3.492 (3) and 3.592 (3) Å, respectively. Inter­molecular C—H⋯O hydrogen bonds and C—H⋯π inter­actions help to stabilize the crystal structure.

## Related literature

The 2,9-dimethyl-1,10-phenanthroline ligand and its substituted derivatives play an important role in the development of coordination chemistry (Kaes *et al.*, 2000 ▶). For related structures of 2,9-dimethyl-1,10-phenanthroline complexes, see: Ding *et al.* (2006 ▶); Harvey *et al.* (2004 ▶); Kaes *et al.* (2000 ▶); Xuan & Zhao (2007 ▶); Zhao & Xuan (2007 ▶). 
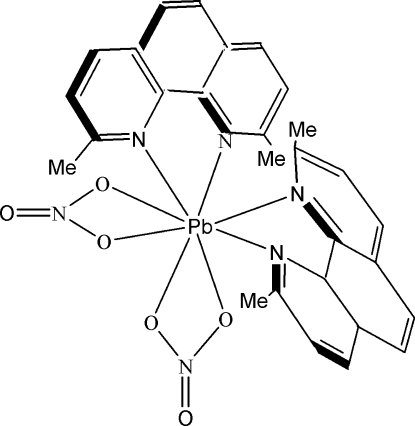


## Experimental

### 

#### Crystal data

[Pb(NO_3_)_2_(C_14_H_12_N_2_)_2_]
*M* *_r_* = 747.73
Orthorhombic, 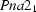

*a* = 19.9164 (4) Å
*b* = 8.0173 (1) Å
*c* = 16.3575 (3) Å
*V* = 2611.90 (8) Å^3^
*Z* = 4
Mo *K*α radiation
μ = 6.52 mm^−1^
*T* = 100 K
0.32 × 0.18 × 0.12 mm

#### Data collection

Oxford Diffraction Gemini-S CCD diffractometer
Absorption correction: multi-scan (*CrysAlis RED*; Oxford Diffraction, 2008 ▶) *T*_min_ = 0.286, *T*_max_ = 0.458
13236 measured reflections
5091 independent reflections
4126 reflections with *I* > 2σ(*I*)
*R*_int_ = 0.030

#### Refinement

*R*[*F* ^2^ > 2σ(*F* ^2^)] = 0.021
*wR*(*F* ^2^) = 0.040
*S* = 0.91
5091 reflections
375 parameters
13 restraints
H-atom parameters constrained
Δρ_max_ = 1.66 e Å^−3^
Δρ_min_ = −0.71 e Å^−3^
Absolute structure: Flack (1983 ▶), 1469 Friedel pairs
Flack parameter: 0.011 (5)

### 

Data collection: *CrysAlis CCD* (Oxford Diffraction, 2008 ▶); cell refinement: *CrysAlis RED* (Oxford Diffraction, 2008 ▶); data reduction: *CrysAlis RED*; program(s) used to solve structure: *SHELXS97* (Sheldrick, 2008 ▶); program(s) used to refine structure: *SHELXL97* (Sheldrick, 2008 ▶); molecular graphics: *PLATON* (Spek, 2009 ▶); software used to prepare material for publication: *PLATON*.

## Supplementary Material

Crystal structure: contains datablocks global, I. DOI: 10.1107/S1600536810022312/xu2779sup1.cif

Structure factors: contains datablocks I. DOI: 10.1107/S1600536810022312/xu2779Isup2.hkl

Additional supplementary materials:  crystallographic information; 3D view; checkCIF report

## Figures and Tables

**Table 1:** Selected bond lengths (Å)

Pb1—O1	2.685 (3)
Pb1—O2	2.703 (4)
Pb1—O4	2.689 (3)
Pb1—O5	2.687 (3)
Pb1—N3	2.702 (3)
Pb1—N4	2.669 (3)
Pb1—N5	2.721 (3)
Pb1—N6	2.712 (3)

**Table 2:** Hydrogen-bond geometry (Å, °)

*D*—H⋯*A*	*D*—H	H⋯*A*	*D*⋯*A*	*D*—H⋯*A*
C13—H1⋯O2	0.98	2.57	3.272 (7)	128
C13—H1⋯O5	0.98	2.53	3.286 (5)	134
C13—H2⋯O4^i^	0.98	2.59	3.496 (5)	153
C10—H5⋯O3^ii^	0.95	2.53	3.192 (5)	127
C8—H6⋯O6^iii^	0.95	2.54	3.376 (5)	147
C4—H9⋯O2^iv^	0.95	2.48	3.401 (6)	163
C14—H10⋯O1	0.98	2.58	3.555 (11)	177
C27—H13⋯O5	0.98	2.35	3.324 (5)	172
C24—H17⋯O6^v^	0.95	2.42	3.158 (5)	135
C19—H20⋯O4^iv^	0.95	2.59	3.482 (6)	156
C28—H22⋯O4	0.98	2.40	3.346 (9)	161
C28—H24⋯O1	0.98	2.55	3.104 (4)	116
C14—H11⋯*Cg*4	0.98	2.77	3.415 (4)	124
C27—H14⋯*Cg*1	0.98	2.69	3.419 (4)	131

Symmetry codes: (i) 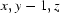; (ii) 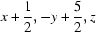; (iii) 
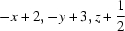
; (iv) 
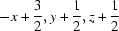
; (v) 
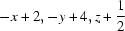
.

**Fig. 1.**
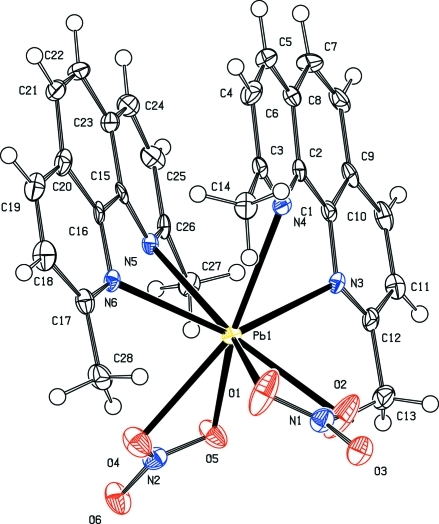
View of the title compound with the atom numbering scheme. Displacement ellipsoids are drawn at the 50% probability level.

**Fig. 2.**
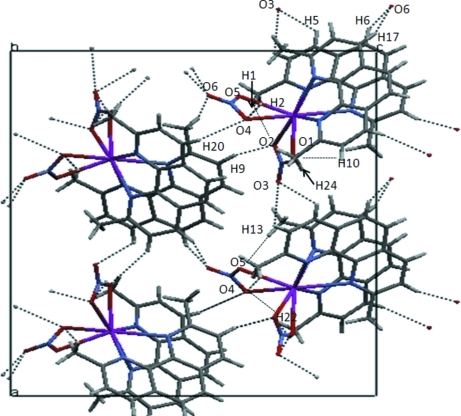
The molecular packing for the title compound, viewed along the b axis. Hydrogen-bonding associations are shown as dotted lines.

## supplementary crystallographic information

### Comment

2,9-Dimethyl-1,10-phenanthroline ligand and its substituted derivates play an important role in the development of coordination chemistry (Kaes *et al.*,2000). There are several reports on coordination of 2,9-dimethyl-1,10-phenanthroline to metals, such as [Co(NO_3_)_2_(C_14_H_12_N_2_)(H_2_O)] (Ding *et al.*, 2006), [Pb(C_14_H_12_N_2_)(C_7_H_5_O_3_)_2_].C_14_H_12_N_2_.H_2_O (Zhao & Xuan, 2007), [Mn(C_7_H_5_O_3_)_2_(C_14_H_12_N_2_)(H_2_O)](C_14_H_12_N_2_.C_2_H_6_O.H_2_O) (Xuan & Zhao, 2007) and [Hg(C_2_H_3_O_2_)_2_(C_14_H_12_N_2_)] (Harvey *et al.*, 2004).

In the structure of the title compound the lead ion is eight-coordinated by four N atoms of two dmphen ligands, four O atoms of two nitrate anions. The resulting coordination is a square antiprismatic geometry, as shown in Fig 1. The Pb—O and Pb—N bond lengths are listed in Table 1. In the crystal structure, there are several C—H···O hydrogen bonds (full details and symmetry codes are given in Table 2 and Fig 2. On the other hand, π-π stacking interactions are between neighboring heteraromatic ring with centroid-centroid distance in the range 3.492 (3)~3.917 (2) Å, the shortest is between *Cg*3 (N5/C15,C23—C26)··· *Cg*5 (C1,C2,C6—C9) is 3.492 (3) Å and dihedral angle between two rings is 7.8 (2)°. In additional, C—H···π interactions [C14 —H11··· *Cg*4 (N6/C16—C20) and C27—H14··· *Cg*1 (N2/C1,C9—C12); full details and symmetry codes are given in Table 2)] are also present.

### Experimental

The mixture of Pb(NO_3_)_2_ (165.5 mg, 0.50 mmole) with dmphen (108.1 mg, 0.50 mmole) in 10 ml H_2_O was seal in a 25 ml stainless-steel reactor with a Teflon linear, heat to 180 °C for 72 hr, and then slowly cooled to room temperature. The pale yellow crystals of the title compound were obtained in 65.85% yield (based on Pb).

### Refinement

H atoms were positioned geometrically with C—H = 0.95 (aromatic) and 0.98 Å (methyl), and refined in the riding-model with U_iso_(H)= 1.5U_eq_(C) for methyl and 1.2U_eq_(C) for the others.

### Crystal data

**Table:**

[Pb(NO_3_)_2_(C_14_H_12_N_2_)_2_]	*F*(000) = 1456
*M**_r_* = 747.73	*D*_x_ = 1.902 Mg m^−^^3^
Orthorhombic, *P**n**a*2_1_	Mo *K*α radiation, λ = 0.71073 Å
Hall symbol: P 2c -2n	Cell parameters from 8515 reflections
*a* = 19.9164 (4) Å	θ = 2.4–29.0°
*b* = 8.0173 (1) Å	µ = 6.52 mm^−^^1^
*c* = 16.3575 (3) Å	*T* = 100 K
*V* = 2611.90 (8) Å^3^	Block, pale yellow
*Z* = 4	0.32 × 0.18 × 0.12 mm

### Data collection

**Table:**

Oxford Diffraction Gemini-S CCD diffractometer	5091 independent reflections
Radiation source: fine-focus sealed tube	4126 reflections with *I* > 2σ(*I*)
graphite	*R*_int_ = 0.030
ω–scan	θ_max_ = 29.1°, θ_min_ = 2.4°
Absorption correction: multi-scan (*CrysAlis RED*; Oxford Diffraction, 2008)	*h* = −26→26
*T*_min_ = 0.286, *T*_max_ = 0.458	*k* = −10→10
13236 measured reflections	*l* = −15→22

### Refinement

**Table:**

Refinement on *F*^2^	Secondary atom site location: difference Fourier map
Least-squares matrix: full	Hydrogen site location: inferred from neighbouring sites
*R*[*F*^2^ > 2σ(*F*^2^)] = 0.021	H-atom parameters constrained
*wR*(*F*^2^) = 0.040	*w* = 1/[σ^2^(*F*_o_^2^) + (0.020*P*)^2^] where *P* = (*F*_o_^2^ + 2*F*_c_^2^)/3
*S* = 0.91	(Δ/σ)_max_ = 0.020
5091 reflections	Δρ_max_ = 1.66 e Å^−^^3^
375 parameters	Δρ_min_ = −0.71 e Å^−^^3^
13 restraints	Absolute structure: Flack (1983), 1469 Friedel pairs
Primary atom site location: structure-invariant direct methods	Flack parameter: 0.011 (5)

### Special details

**Table:**

Geometry. Bond distances, angles *etc*. have been calculated using the rounded fractional coordinates. All su's are estimated from the variances of the (full) variance-covariance matrix. The cell e.s.d.'s are taken into account in the estimation of distances, angles and torsion angles
Refinement. Refinement on *F*^2^ for ALL reflections except those flagged by the user for potential systematic errors. Weighted *R*-factors *wR* and all goodnesses of fit *S* are based on *F*^2^, conventional *R*-factors *R* are based on *F*, with *F* set to zero for negative *F*^2^. The observed criterion of *F*^2^ > σ(*F*^2^) is used only for calculating -*R*-factor-obs *etc*. and is not relevant to the choice of reflections for refinement. *R*-factors based on *F*^2^ are statistically about twice as large as those based on *F*, and *R*-factors based on ALL data will be even larger.

### Fractional atomic coordinates and isotropic or equivalent isotropic displacement parameters (Å^2^)

**Table:**

	*x*	*y*	*z*	*U*_iso_*/*U*_eq_	
Pb1	0.81279 (1)	0.68918 (1)	0.27495 (2)	0.0123 (1)	
O1	0.67839 (13)	0.6637 (3)	0.2709 (6)	0.0430 (12)	
O2	0.72791 (16)	0.4501 (5)	0.2212 (3)	0.0459 (13)	
O3	0.61990 (14)	0.4502 (4)	0.2321 (2)	0.0248 (10)	
O4	0.80174 (17)	0.8914 (4)	0.1445 (2)	0.0298 (11)	
O5	0.86851 (17)	0.6812 (4)	0.12537 (19)	0.0270 (11)	
O6	0.87017 (17)	0.8896 (4)	0.0410 (2)	0.0289 (11)	
N1	0.67479 (17)	0.5206 (4)	0.2413 (2)	0.0202 (11)	
N2	0.84797 (18)	0.8229 (5)	0.1027 (2)	0.0190 (12)	
N3	0.88824 (16)	0.4171 (4)	0.3076 (2)	0.0127 (10)	
N4	0.82737 (17)	0.5960 (4)	0.4306 (2)	0.0134 (11)	
N5	0.92384 (17)	0.8515 (4)	0.3302 (2)	0.0129 (10)	
N6	0.79763 (16)	0.9939 (4)	0.3445 (2)	0.0132 (11)	
C1	0.9218 (2)	0.4374 (5)	0.3792 (3)	0.0136 (11)	
C2	0.8902 (2)	0.5331 (5)	0.4438 (3)	0.0147 (12)	
C3	0.7958 (2)	0.6729 (5)	0.4913 (3)	0.0170 (14)	
C4	0.8267 (2)	0.6970 (5)	0.5682 (3)	0.0213 (14)	
C5	0.8905 (2)	0.6395 (5)	0.5801 (3)	0.0230 (14)	
C6	0.9239 (2)	0.5531 (5)	0.5186 (3)	0.0173 (12)	
C7	0.9892 (3)	0.4878 (9)	0.5286 (5)	0.0233 (18)	
C8	1.0189 (2)	0.3935 (5)	0.4696 (3)	0.0210 (14)	
C9	0.9856 (2)	0.3655 (5)	0.3941 (3)	0.0160 (12)	
C10	1.0119 (2)	0.2629 (5)	0.3315 (3)	0.0197 (14)	
C11	0.9761 (2)	0.2353 (4)	0.2627 (4)	0.0169 (19)	
C12	0.9135 (2)	0.3154 (5)	0.2516 (2)	0.0168 (11)	
C13	0.8733 (3)	0.2845 (5)	0.1756 (3)	0.0227 (17)	
C14	0.7256 (2)	0.7309 (5)	0.4779 (3)	0.0210 (16)	
C15	0.9087 (2)	0.9389 (5)	0.3990 (3)	0.0132 (11)	
C16	0.8436 (2)	1.0192 (5)	0.4054 (3)	0.0140 (11)	
C17	0.7408 (2)	1.0801 (5)	0.3454 (3)	0.0170 (12)	
C18	0.7262 (2)	1.1946 (6)	0.4074 (3)	0.0233 (14)	
C19	0.7695 (2)	1.2141 (5)	0.4706 (3)	0.0220 (16)	
C20	0.8303 (2)	1.1234 (5)	0.4725 (3)	0.0197 (16)	
C21	0.8778 (3)	1.1361 (5)	0.5382 (3)	0.0220 (14)	
C22	0.9370 (3)	1.0579 (5)	0.5340 (3)	0.0240 (14)	
C23	0.9550 (2)	0.9622 (5)	0.4634 (3)	0.0190 (14)	
C24	1.0187 (2)	0.8927 (5)	0.4539 (3)	0.0220 (16)	
C25	1.0347 (2)	0.8135 (5)	0.3827 (3)	0.0210 (14)	
C26	0.9861 (2)	0.7931 (5)	0.3218 (3)	0.0147 (12)	
C27	1.0043 (2)	0.7101 (5)	0.2427 (3)	0.0173 (12)	
C28	0.69100 (18)	1.0494 (4)	0.2774 (7)	0.0222 (10)	
H1	0.84510	0.38190	0.16410	0.0340*	
H2	0.84480	0.18630	0.18350	0.0340*	
H3	0.90380	0.26520	0.12950	0.0340*	
H4	0.99300	0.16230	0.22180	0.0200*	
H5	1.05490	0.21320	0.33790	0.0230*	
H6	1.06200	0.34620	0.47900	0.0250*	
H7	1.01310	0.51040	0.57770	0.0280*	
H8	0.91240	0.65860	0.63090	0.0270*	
H9	0.80340	0.75240	0.61100	0.0260*	
H10	0.71350	0.71560	0.42030	0.0310*	
H11	0.72210	0.84920	0.49220	0.0310*	
H12	0.69500	0.66590	0.51230	0.0310*	
H13	0.96550	0.71240	0.20580	0.0260*	
H14	1.01730	0.59420	0.25320	0.0260*	
H15	1.04190	0.76950	0.21740	0.0260*	
H16	1.07890	0.77200	0.37450	0.0250*	
H17	1.05080	0.90050	0.49670	0.0270*	
H18	0.96750	1.06590	0.57850	0.0290*	
H19	0.86700	1.20060	0.58510	0.0260*	
H20	0.75900	1.28900	0.51370	0.0270*	
H21	0.68610	1.25850	0.40520	0.0280*	
H22	0.71420	1.00010	0.23040	0.0330*	
H23	0.67040	1.15530	0.26110	0.0330*	
H24	0.65600	0.97270	0.29650	0.0330*	

### Atomic displacement parameters (Å^2^)

**Table:**

	*U*^11^	*U*^22^	*U*^33^	*U*^12^	*U*^13^	*U*^23^
Pb1	0.0120 (1)	0.0136 (1)	0.0112 (1)	−0.0004 (1)	−0.0004 (2)	−0.0012 (2)
O1	0.0227 (15)	0.0283 (15)	0.078 (3)	−0.0031 (12)	0.010 (3)	−0.026 (3)
O2	0.0156 (18)	0.041 (2)	0.081 (3)	−0.0035 (16)	0.0004 (19)	−0.036 (2)
O3	0.0145 (16)	0.0271 (17)	0.0329 (18)	−0.0074 (13)	0.0005 (15)	−0.0012 (15)
O4	0.036 (2)	0.0275 (19)	0.026 (2)	0.0107 (15)	0.0072 (17)	0.0043 (16)
O5	0.035 (2)	0.0249 (18)	0.0212 (18)	0.0146 (17)	0.0073 (16)	0.0059 (16)
O6	0.036 (2)	0.0262 (18)	0.0245 (19)	−0.0001 (15)	0.0157 (17)	0.0075 (16)
N1	0.015 (2)	0.0205 (18)	0.025 (2)	0.0011 (14)	−0.0044 (16)	−0.0020 (15)
N2	0.019 (2)	0.022 (2)	0.016 (2)	−0.0018 (18)	0.0026 (17)	−0.0026 (19)
N3	0.0139 (18)	0.0095 (17)	0.0148 (18)	−0.0008 (14)	0.0024 (14)	−0.0023 (14)
N4	0.014 (2)	0.0145 (19)	0.0117 (19)	−0.0053 (15)	0.0022 (16)	0.0006 (16)
N5	0.0123 (18)	0.0112 (17)	0.0152 (19)	−0.0040 (13)	0.0022 (16)	0.0021 (14)
N6	0.0154 (19)	0.0103 (17)	0.0140 (19)	−0.0031 (13)	0.0055 (16)	−0.0010 (14)
C1	0.013 (2)	0.0107 (19)	0.017 (2)	−0.0035 (16)	−0.0004 (18)	0.0052 (18)
C2	0.018 (2)	0.009 (2)	0.017 (2)	−0.0054 (16)	0.001 (2)	0.0047 (17)
C3	0.026 (3)	0.010 (2)	0.015 (2)	−0.0037 (17)	0.0009 (19)	0.0038 (18)
C4	0.030 (3)	0.018 (2)	0.016 (2)	−0.006 (2)	0.006 (2)	−0.005 (2)
C5	0.036 (3)	0.018 (2)	0.015 (2)	−0.006 (2)	−0.004 (2)	−0.0011 (19)
C6	0.024 (2)	0.014 (2)	0.014 (2)	−0.0049 (18)	−0.001 (2)	0.0050 (18)
C7	0.017 (4)	0.028 (3)	0.025 (2)	−0.004 (3)	−0.016 (3)	0.004 (2)
C8	0.018 (2)	0.018 (2)	0.027 (3)	0.0011 (18)	−0.007 (2)	0.011 (2)
C9	0.014 (2)	0.013 (2)	0.021 (2)	−0.0027 (16)	0.004 (2)	0.0091 (18)
C10	0.015 (2)	0.013 (2)	0.031 (3)	0.0048 (16)	0.005 (2)	0.0071 (19)
C11	0.018 (2)	0.0147 (18)	0.018 (5)	0.0016 (14)	0.008 (2)	0.0014 (19)
C12	0.020 (2)	0.0125 (18)	0.018 (2)	−0.0080 (18)	0.0029 (17)	0.0019 (19)
C13	0.031 (3)	0.018 (3)	0.019 (3)	−0.002 (2)	0.002 (2)	−0.005 (2)
C14	0.026 (3)	0.019 (2)	0.018 (3)	−0.0002 (18)	0.011 (2)	−0.0016 (19)
C15	0.015 (2)	0.0085 (19)	0.016 (2)	−0.0037 (16)	0.0011 (19)	0.0079 (18)
C16	0.020 (2)	0.0061 (19)	0.016 (2)	−0.0048 (16)	0.005 (2)	0.0034 (17)
C17	0.017 (2)	0.014 (2)	0.020 (2)	−0.0009 (18)	0.006 (2)	0.0031 (19)
C18	0.021 (2)	0.019 (2)	0.030 (3)	0.003 (2)	0.007 (2)	−0.001 (2)
C19	0.031 (3)	0.016 (2)	0.019 (3)	−0.0051 (19)	0.013 (2)	−0.0041 (19)
C20	0.025 (3)	0.013 (2)	0.021 (3)	−0.0071 (17)	0.008 (2)	−0.0013 (19)
C21	0.036 (3)	0.017 (2)	0.013 (2)	−0.010 (2)	0.000 (2)	−0.0008 (18)
C22	0.035 (3)	0.022 (2)	0.015 (2)	−0.013 (2)	−0.009 (2)	0.002 (2)
C23	0.025 (3)	0.016 (2)	0.016 (2)	−0.0101 (18)	−0.003 (2)	0.0034 (18)
C24	0.020 (3)	0.022 (2)	0.024 (3)	−0.0088 (19)	−0.008 (2)	0.006 (2)
C25	0.015 (2)	0.025 (2)	0.023 (3)	−0.003 (2)	−0.002 (2)	0.008 (2)
C26	0.015 (2)	0.008 (2)	0.021 (2)	−0.0051 (18)	0.003 (2)	0.003 (2)
C27	0.012 (2)	0.019 (2)	0.021 (2)	−0.0039 (19)	0.0028 (19)	0.000 (2)
C28	0.0195 (17)	0.0201 (16)	0.0269 (19)	0.0059 (15)	−0.003 (4)	0.004 (5)

### Geometric parameters (Å, °)

**Table:**

Pb1—O1	2.685 (3)	C15—C23	1.412 (6)
Pb1—O2	2.703 (4)	C16—C20	1.405 (6)
Pb1—O4	2.689 (3)	C17—C18	1.399 (7)
Pb1—O5	2.687 (3)	C17—C28	1.511 (10)
Pb1—N3	2.702 (3)	C18—C19	1.355 (6)
Pb1—N4	2.669 (3)	C19—C20	1.413 (6)
Pb1—N5	2.721 (3)	C20—C21	1.435 (7)
Pb1—N6	2.712 (3)	C21—C22	1.337 (8)
O1—N1	1.247 (5)	C22—C23	1.432 (7)
O2—N1	1.244 (5)	C23—C24	1.394 (6)
O3—N1	1.240 (4)	C24—C25	1.364 (7)
O4—N2	1.272 (5)	C25—C26	1.399 (6)
O5—N2	1.263 (5)	C26—C27	1.499 (7)
O6—N2	1.225 (5)	C4—H9	0.9500
N3—C1	1.358 (6)	C5—H8	0.9500
N3—C12	1.326 (5)	C7—H7	0.9500
N4—C2	1.366 (5)	C8—H6	0.9500
N4—C3	1.327 (6)	C10—H5	0.9500
N5—C15	1.360 (6)	C11—H4	0.9500
N5—C26	1.333 (5)	C13—H1	0.9800
N6—C16	1.368 (6)	C13—H2	0.9800
N6—C17	1.326 (5)	C13—H3	0.9800
C1—C2	1.450 (6)	C14—H10	0.9800
C1—C9	1.416 (6)	C14—H11	0.9800
C2—C6	1.405 (7)	C14—H12	0.9800
C3—C4	1.414 (7)	C18—H21	0.9500
C3—C14	1.490 (6)	C19—H20	0.9500
C4—C5	1.366 (6)	C21—H19	0.9500
C5—C6	1.391 (6)	C22—H18	0.9500
C6—C7	1.412 (7)	C24—H17	0.9500
C7—C8	1.361 (9)	C25—H16	0.9500
C8—C9	1.420 (7)	C27—H13	0.9800
C9—C10	1.414 (6)	C27—H14	0.9800
C10—C11	1.351 (7)	C27—H15	0.9800
C11—C12	1.414 (6)	C28—H22	0.9800
C12—C13	1.499 (6)	C28—H23	0.9800
C15—C16	1.451 (6)	C28—H24	0.9800
			
Pb1···H1	3.1300	C14···C16	3.503 (6)
Pb1···H10	3.1000	C15···C11^vii^	3.524 (7)
Pb1···H13	3.2500	C15···C10^vii^	3.492 (6)
Pb1···H22	3.2600	C15···C3	3.447 (6)
O1···O2	2.137 (6)	C15···C2	3.355 (6)
O1···C28	3.104 (4)	C16···C3	3.254 (6)
O1···C25^i^	3.401 (7)	C16···C14	3.503 (6)
O2···C4^ii^	3.401 (6)	C17···C14	3.554 (6)
O2···C13	3.272 (7)	C18···O6^ix^	3.302 (6)
O2···O1	2.137 (6)	C19···N2^ix^	3.302 (6)
O3···C11^iii^	3.266 (5)	C19···O6^ix^	3.323 (5)
O3···C10^iii^	3.192 (5)	C19···N4^vii^	3.336 (5)
O4···O5	2.169 (5)	C19···C2^vii^	3.537 (6)
O4···C28	3.346 (9)	C20···C2^vii^	3.526 (6)
O5···C13	3.286 (5)	C20···C1^vii^	3.462 (6)
O5···C27	3.324 (5)	C21···C6^vii^	3.482 (6)
O5···O4	2.169 (5)	C21···C7^vii^	3.591 (8)
O6···C19^ii^	3.323 (5)	C21···C2^vii^	3.546 (6)
O6···C8^iv^	3.376 (5)	C22···C8^vii^	3.318 (6)
O6···C18^ii^	3.302 (6)	C22···C9^vii^	3.501 (6)
O6···C24^v^	3.158 (5)	C22···C5	3.561 (6)
O1···H16^i^	2.6600	C23···C10^vii^	3.428 (6)
O1···H24	2.5500	C23···C6	3.458 (6)
O1···H15^i^	2.9100	C23···C5	3.462 (6)
O1···H22	2.8700	C23···C9^vii^	3.480 (6)
O1···H10	2.5800	C24···C6	3.478 (6)
O2···H23^vi^	2.7100	C24···C10^vii^	3.583 (6)
O2···H1	2.5700	C24···C7	3.518 (8)
O2···H9^ii^	2.4800	C24···O6^xii^	3.158 (5)
O3···H15^i^	2.7400	C25···O1^xiii^	3.401 (7)
O3···H5^iii^	2.5300	C26···C1	3.264 (6)
O3···H4^iii^	2.6900	C26···C2	3.461 (6)
O3···H23^vi^	2.6100	C27···O5	3.324 (5)
O4···H22	2.4000	C27···C1	3.531 (6)
O4···H20^ii^	2.5900	C28···O1	3.104 (4)
O4···H2^vii^	2.5900	C28···O4	3.346 (9)
O5···H6^iv^	2.7700	C1···H14	3.0700
O5···H13	2.3500	C9···H14	3.0100
O5···H1	2.5300	C10···H14	2.9500
O6···H17^v^	2.4200	C11···H14	3.0000
O6···H6^iv^	2.5400	C12···H14	3.0400
O6···H12^viii^	2.6100	C17···H11	3.0500
O6···H20^ii^	2.7300	C18···H11	3.1000
O6···H21^ii^	2.7000	C19···H11	3.0900
N2···C19^ii^	3.302 (6)	C20···H11	3.1000
N3···N4	2.752 (5)	H1···Pb1	3.1300
N3···C2	2.415 (6)	H1···O2	2.5700
N4···C16	3.433 (5)	H1···O5	2.5300
N4···C1	2.421 (5)	H2···O4^vi^	2.5900
N4···N3	2.752 (5)	H3···H4	2.4700
N4···N5	3.253 (5)	H3···H7^iv^	2.5900
N4···C19^vi^	3.336 (5)	H4···H3	2.4700
N4···C15	3.232 (5)	H4···O3^xi^	2.6900
N5···C16	2.424 (5)	H5···H6	2.5500
N5···C11^vii^	3.431 (5)	H5···O3^xi^	2.5300
N5···N4	3.253 (5)	H6···H5	2.5500
N5···N6	2.771 (5)	H6···O5^x^	2.7700
N5···C1	3.416 (5)	H6···O6^x^	2.5400
N5···C2	3.228 (5)	H7···H8	2.4900
N6···C14	3.356 (5)	H7···H3^x^	2.5900
N6···N5	2.771 (5)	H8···H7	2.4900
N6···C15	2.425 (5)	H9···O2^ix^	2.4800
N1···H23^vi^	2.9500	H10···Pb1	3.1000
N2···H20^ii^	2.5900	H10···O1	2.5800
C1···C20^vi^	3.462 (6)	H11···C17	3.0500
C1···C26	3.264 (6)	H11···C18	3.1000
C1···C27	3.531 (6)	H11···C19	3.0900
C2···C20^vi^	3.526 (6)	H11···C20	3.1000
C2···C15	3.355 (6)	H12···O6^xiv^	2.6100
C2···C19^vi^	3.537 (6)	H13···Pb1	3.2500
C2···C21^vi^	3.546 (6)	H13···O5	2.3500
C2···C26	3.461 (6)	H14···C1	3.0700
C3···C16	3.254 (6)	H14···C9	3.0100
C3···C15	3.447 (6)	H14···C10	2.9500
C4···O2^ix^	3.401 (6)	H14···C11	3.0000
C5···C23	3.462 (6)	H14···C12	3.0400
C5···C22	3.561 (6)	H15···O1^xiii^	2.9100
C6···C24	3.478 (6)	H15···O3^xiii^	2.7400
C6···C21^vi^	3.482 (6)	H16···O1^xiii^	2.6600
C6···C23	3.458 (6)	H17···H18	2.5100
C7···C24	3.518 (8)	H17···O6^xii^	2.4200
C7···C21^vi^	3.591 (8)	H18···H17	2.5100
C8···C22^vi^	3.318 (6)	H19···H20	2.5500
C8···O6^x^	3.376 (5)	H20···H19	2.5500
C9···C22^vi^	3.501 (6)	H20···O4^ix^	2.5900
C9···C23^vi^	3.480 (6)	H20···O6^ix^	2.7300
C10···C23^vi^	3.428 (6)	H20···N2^ix^	2.5900
C10···C24^vi^	3.583 (6)	H21···H23	2.5200
C10···C15^vi^	3.492 (6)	H21···O6^ix^	2.7000
C10···O3^xi^	3.192 (5)	H22···Pb1	3.2600
C11···C15^vi^	3.524 (7)	H22···O1	2.8700
C11···O3^xi^	3.266 (5)	H22···O4	2.4000
C11···N5^vi^	3.431 (5)	H23···O2^vii^	2.7100
C13···O2	3.272 (7)	H23···O3^vii^	2.6100
C13···O5	3.286 (5)	H23···N1^vii^	2.9500
C14···C17	3.554 (6)	H23···H21	2.5200
C14···N6	3.356 (5)	H24···O1	2.5500
			
O1—Pb1—O2	46.73 (13)	C11—C12—C13	120.2 (4)
O1—Pb1—O4	86.83 (18)	N5—C15—C16	119.1 (4)
O1—Pb1—O5	112.8 (2)	N5—C15—C23	122.7 (4)
O1—Pb1—N3	119.85 (10)	C16—C15—C23	118.1 (4)
O1—Pb1—N4	96.4 (2)	N6—C16—C15	118.7 (4)
O1—Pb1—N5	148.75 (15)	N6—C16—C20	122.1 (4)
O1—Pb1—N6	88.16 (12)	C15—C16—C20	119.3 (4)
O2—Pb1—O4	96.79 (12)	N6—C17—C18	121.8 (4)
O2—Pb1—O5	86.86 (12)	N6—C17—C28	117.9 (4)
O2—Pb1—N3	80.77 (10)	C18—C17—C28	120.3 (4)
O2—Pb1—N4	100.36 (12)	C17—C18—C19	119.8 (4)
O2—Pb1—N5	162.89 (10)	C18—C19—C20	120.2 (4)
O2—Pb1—N6	134.89 (10)	C16—C20—C19	116.8 (4)
O4—Pb1—O5	47.60 (10)	C16—C20—C21	120.2 (4)
O4—Pb1—N3	133.56 (10)	C19—C20—C21	123.0 (4)
O4—Pb1—N4	159.17 (10)	C20—C21—C22	120.6 (4)
O4—Pb1—N5	92.39 (10)	C21—C22—C23	120.9 (5)
O4—Pb1—N6	77.33 (10)	C15—C23—C22	120.6 (4)
O5—Pb1—N3	86.05 (10)	C15—C23—C24	117.3 (4)
O5—Pb1—N4	144.77 (10)	C22—C23—C24	122.1 (4)
O5—Pb1—N5	88.75 (10)	C23—C24—C25	119.6 (4)
O5—Pb1—N6	116.70 (10)	C24—C25—C26	120.1 (4)
N3—Pb1—N4	61.64 (10)	N5—C26—C25	122.0 (4)
N3—Pb1—N5	82.44 (10)	N5—C26—C27	118.0 (4)
N3—Pb1—N6	134.93 (10)	C25—C26—C27	120.0 (4)
N4—Pb1—N5	74.25 (10)	C3—C4—H9	120.00
N4—Pb1—N6	82.19 (10)	C5—C4—H9	120.00
N5—Pb1—N6	61.33 (10)	C4—C5—H8	120.00
Pb1—O1—N1	97.9 (2)	C6—C5—H8	120.00
Pb1—O2—N1	97.1 (3)	C6—C7—H7	119.00
Pb1—O4—N2	96.1 (2)	C8—C7—H7	119.00
Pb1—O5—N2	96.4 (2)	C7—C8—H6	120.00
O1—N1—O2	118.2 (3)	C9—C8—H6	120.00
O1—N1—O3	121.1 (3)	C9—C10—H5	120.00
O2—N1—O3	120.7 (3)	C11—C10—H5	120.00
O4—N2—O5	117.7 (3)	C10—C11—H4	120.00
O4—N2—O6	121.1 (4)	C12—C11—H4	120.00
O5—N2—O6	121.2 (4)	C12—C13—H1	109.00
Pb1—N3—C1	110.3 (2)	C12—C13—H2	109.00
Pb1—N3—C12	124.8 (2)	C12—C13—H3	109.00
C1—N3—C12	118.9 (3)	H1—C13—H2	109.00
Pb1—N4—C2	110.7 (3)	H1—C13—H3	109.00
Pb1—N4—C3	122.2 (3)	H2—C13—H3	109.00
C2—N4—C3	119.2 (4)	C3—C14—H10	109.00
Pb1—N5—C15	110.0 (2)	C3—C14—H11	110.00
Pb1—N5—C26	123.7 (3)	C3—C14—H12	109.00
C15—N5—C26	118.2 (4)	H10—C14—H11	109.00
Pb1—N6—C16	111.4 (2)	H10—C14—H12	109.00
Pb1—N6—C17	124.7 (3)	H11—C14—H12	109.00
C16—N6—C17	119.1 (4)	C17—C18—H21	120.00
N3—C1—C2	118.6 (4)	C19—C18—H21	120.00
N3—C1—C9	122.8 (4)	C18—C19—H20	120.00
C2—C1—C9	118.7 (4)	C20—C19—H20	120.00
N4—C2—C1	118.5 (4)	C20—C21—H19	120.00
N4—C2—C6	122.2 (4)	C22—C21—H19	120.00
C1—C2—C6	119.2 (4)	C21—C22—H18	120.00
N4—C3—C4	121.5 (4)	C23—C22—H18	120.00
N4—C3—C14	118.7 (4)	C23—C24—H17	120.00
C4—C3—C14	119.8 (4)	C25—C24—H17	120.00
C3—C4—C5	119.1 (4)	C24—C25—H16	120.00
C4—C5—C6	120.7 (4)	C26—C25—H16	120.00
C2—C6—C5	117.3 (4)	C26—C27—H13	109.00
C2—C6—C7	119.9 (5)	C26—C27—H14	109.00
C5—C6—C7	122.8 (5)	C26—C27—H15	109.00
C6—C7—C8	121.6 (6)	H13—C27—H14	110.00
C7—C8—C9	120.1 (4)	H13—C27—H15	109.00
C1—C9—C8	120.3 (4)	H14—C27—H15	109.00
C1—C9—C10	116.4 (4)	C17—C28—H22	109.00
C8—C9—C10	123.3 (4)	C17—C28—H23	110.00
C9—C10—C11	120.2 (4)	C17—C28—H24	110.00
C10—C11—C12	119.9 (4)	H22—C28—H23	109.00
N3—C12—C11	121.7 (4)	H22—C28—H24	109.00
N3—C12—C13	118.2 (4)	H23—C28—H24	109.00
			
O2—Pb1—O1—N1	2.2 (3)	Pb1—O5—N2—O6	167.8 (3)
O4—Pb1—O1—N1	104.6 (5)	Pb1—N3—C1—C2	33.0 (4)
O5—Pb1—O1—N1	63.7 (5)	Pb1—N3—C1—C9	−148.7 (3)
N3—Pb1—O1—N1	−35.2 (6)	C12—N3—C1—C2	−172.9 (4)
N4—Pb1—O1—N1	−96.1 (5)	C12—N3—C1—C9	5.5 (6)
N5—Pb1—O1—N1	−166.0 (3)	Pb1—N3—C12—C11	146.5 (3)
N6—Pb1—O1—N1	−178.0 (5)	Pb1—N3—C12—C13	−34.4 (5)
O1—Pb1—O2—N1	−2.2 (3)	C1—N3—C12—C11	−3.6 (6)
O4—Pb1—O2—N1	−81.3 (3)	C1—N3—C12—C13	175.5 (4)
O5—Pb1—O2—N1	−128.0 (3)	Pb1—N4—C2—C1	−35.5 (4)
N3—Pb1—O2—N1	145.5 (3)	Pb1—N4—C2—C6	146.8 (3)
N4—Pb1—O2—N1	86.8 (3)	C3—N4—C2—C1	174.9 (4)
N6—Pb1—O2—N1	−2.5 (4)	C3—N4—C2—C6	−2.8 (6)
O1—Pb1—O4—N2	−133.4 (3)	Pb1—N4—C3—C4	−143.4 (3)
O2—Pb1—O4—N2	−87.6 (2)	Pb1—N4—C3—C14	38.1 (5)
O5—Pb1—O4—N2	−8.2 (2)	C2—N4—C3—C4	2.6 (6)
N3—Pb1—O4—N2	−3.9 (3)	C2—N4—C3—C14	−176.0 (4)
N4—Pb1—O4—N2	127.1 (3)	Pb1—N5—C15—C16	−37.0 (4)
N5—Pb1—O4—N2	77.9 (2)	Pb1—N5—C15—C23	146.2 (3)
N6—Pb1—O4—N2	137.8 (2)	C26—N5—C15—C16	173.3 (4)
O1—Pb1—O5—N2	70.6 (2)	C26—N5—C15—C23	−3.5 (6)
O2—Pb1—O5—N2	110.4 (2)	Pb1—N5—C26—C25	−142.7 (3)
O4—Pb1—O5—N2	8.3 (2)	Pb1—N5—C26—C27	40.0 (5)
N3—Pb1—O5—N2	−168.6 (2)	C15—N5—C26—C25	2.6 (6)
N4—Pb1—O5—N2	−146.0 (2)	C15—N5—C26—C27	−174.7 (4)
N5—Pb1—O5—N2	−86.1 (2)	Pb1—N6—C16—C15	30.5 (4)
N6—Pb1—O5—N2	−29.4 (3)	Pb1—N6—C16—C20	−151.2 (3)
O1—Pb1—N3—C1	−115.3 (3)	C17—N6—C16—C15	−173.1 (4)
O1—Pb1—N3—C12	92.4 (4)	C17—N6—C16—C20	5.3 (6)
O2—Pb1—N3—C1	−142.0 (3)	Pb1—N6—C17—C18	152.3 (3)
O2—Pb1—N3—C12	65.8 (3)	Pb1—N6—C17—C28	−26.9 (6)
O4—Pb1—N3—C1	127.4 (3)	C16—N6—C17—C18	−0.8 (6)
O4—Pb1—N3—C12	−24.8 (4)	C16—N6—C17—C28	−180.0 (4)
O5—Pb1—N3—C1	130.6 (3)	N3—C1—C2—N4	1.3 (6)
O5—Pb1—N3—C12	−21.7 (3)	N3—C1—C2—C6	179.1 (4)
N4—Pb1—N3—C1	−34.8 (3)	C9—C1—C2—N4	−177.1 (4)
N4—Pb1—N3—C12	173.0 (3)	C9—C1—C2—C6	0.7 (6)
N5—Pb1—N3—C1	41.3 (3)	N3—C1—C9—C8	178.8 (4)
N5—Pb1—N3—C12	−110.9 (3)	N3—C1—C9—C10	−3.1 (6)
N6—Pb1—N3—C1	6.0 (3)	C2—C1—C9—C8	−2.8 (6)
N6—Pb1—N3—C12	−146.2 (3)	C2—C1—C9—C10	175.3 (4)
O1—Pb1—N4—C2	156.1 (3)	N4—C2—C6—C5	0.5 (6)
O1—Pb1—N4—C3	−55.4 (3)	N4—C2—C6—C7	−179.0 (5)
O2—Pb1—N4—C2	109.0 (3)	C1—C2—C6—C5	−177.2 (4)
O2—Pb1—N4—C3	−102.5 (3)	C1—C2—C6—C7	3.3 (7)
O4—Pb1—N4—C2	−106.1 (3)	N4—C3—C4—C5	−0.1 (6)
O4—Pb1—N4—C3	42.4 (5)	C14—C3—C4—C5	178.4 (4)
O5—Pb1—N4—C2	9.6 (3)	C3—C4—C5—C6	−2.3 (6)
O5—Pb1—N4—C3	158.1 (3)	C4—C5—C6—C2	2.1 (6)
N3—Pb1—N4—C2	35.5 (2)	C4—C5—C6—C7	−178.5 (5)
N3—Pb1—N4—C3	−176.0 (3)	C2—C6—C7—C8	−5.3 (9)
N5—Pb1—N4—C2	−54.4 (3)	C5—C6—C7—C8	175.2 (5)
N5—Pb1—N4—C3	94.1 (3)	C6—C7—C8—C9	3.2 (9)
N6—Pb1—N4—C2	−116.7 (3)	C7—C8—C9—C1	0.9 (7)
N6—Pb1—N4—C3	31.8 (3)	C7—C8—C9—C10	−177.0 (5)
O1—Pb1—N5—C15	21.7 (5)	C1—C9—C10—C11	−1.2 (6)
O1—Pb1—N5—C26	169.5 (4)	C8—C9—C10—C11	176.9 (4)
O4—Pb1—N5—C15	109.5 (3)	C9—C10—C11—C12	2.9 (6)
O4—Pb1—N5—C26	−102.7 (3)	C10—C11—C12—N3	−0.5 (6)
O5—Pb1—N5—C15	157.0 (3)	C10—C11—C12—C13	−179.6 (4)
O5—Pb1—N5—C26	−55.3 (3)	N5—C15—C16—N6	4.8 (6)
N3—Pb1—N5—C15	−116.8 (3)	N5—C15—C16—C20	−173.6 (4)
N3—Pb1—N5—C26	30.9 (3)	C23—C15—C16—N6	−178.2 (4)
N4—Pb1—N5—C15	−54.3 (3)	C23—C15—C16—C20	3.4 (6)
N4—Pb1—N5—C26	93.5 (3)	N5—C15—C23—C22	178.9 (4)
N6—Pb1—N5—C15	35.4 (3)	N5—C15—C23—C24	0.7 (6)
N6—Pb1—N5—C26	−176.9 (4)	C16—C15—C23—C22	2.0 (6)
O1—Pb1—N6—C16	139.5 (3)	C16—C15—C23—C24	−176.2 (4)
O1—Pb1—N6—C17	−15.4 (4)	N6—C16—C20—C19	−5.9 (6)
O2—Pb1—N6—C16	139.7 (3)	N6—C16—C20—C21	175.1 (4)
O2—Pb1—N6—C17	−15.2 (4)	C15—C16—C20—C19	172.5 (4)
O4—Pb1—N6—C16	−133.3 (3)	C15—C16—C20—C21	−6.6 (6)
O4—Pb1—N6—C17	71.8 (3)	N6—C17—C18—C19	−2.9 (7)
O5—Pb1—N6—C16	−105.8 (3)	C28—C17—C18—C19	176.2 (5)
O5—Pb1—N6—C17	99.3 (3)	C17—C18—C19—C20	2.2 (7)
N3—Pb1—N6—C16	7.4 (3)	C18—C19—C20—C16	2.0 (6)
N3—Pb1—N6—C17	−147.5 (3)	C18—C19—C20—C21	−178.9 (4)
N4—Pb1—N6—C16	42.9 (3)	C16—C20—C21—C22	4.4 (7)
N4—Pb1—N6—C17	−112.0 (3)	C19—C20—C21—C22	−174.6 (4)
N5—Pb1—N6—C16	−33.4 (3)	C20—C21—C22—C23	1.2 (7)
N5—Pb1—N6—C17	171.7 (4)	C21—C22—C23—C15	−4.4 (7)
Pb1—O1—N1—O2	−4.0 (6)	C21—C22—C23—C24	173.7 (4)
Pb1—O1—N1—O3	176.0 (3)	C15—C23—C24—C25	3.1 (6)
Pb1—O2—N1—O1	3.9 (6)	C22—C23—C24—C25	−175.1 (4)
Pb1—O2—N1—O3	−176.1 (3)	C23—C24—C25—C26	−4.0 (6)
Pb1—O4—N2—O5	14.7 (4)	C24—C25—C26—N5	1.1 (6)
Pb1—O4—N2—O6	−167.8 (3)	C24—C25—C26—C27	178.4 (4)
Pb1—O5—N2—O4	−14.7 (4)		

Symmetry codes: (i) *x*−1/2, −*y*+7/2, *z*; (ii) −*x*+3/2, *y*−1/2, *z*−1/2; (iii) *x*−1/2, −*y*+5/2, *z*; (iv) −*x*+2, −*y*+3, *z*−1/2; (v) −*x*+2, −*y*+4, *z*−1/2; (vi) *x*, *y*−1, *z*; (vii) *x*, *y*+1, *z*; (viii) −*x*+3/2, *y*+1/2, *z*−1/2; (ix) −*x*+3/2, *y*+1/2, *z*+1/2; (x) −*x*+2, −*y*+3, *z*+1/2; (xi) *x*+1/2, −*y*+5/2, *z*; (xii) −*x*+2, −*y*+4, *z*+1/2; (xiii) *x*+1/2, −*y*+7/2, *z*; (xiv) −*x*+3/2, *y*−1/2, *z*+1/2.

### Hydrogen-bond geometry (Å, °)

**Table:**

*D*—H···*A*	*D*—H	H···*A*	*D*···*A*	*D*—H···*A*
C13—H1···O2	0.98	2.57	3.272 (7)	128
C13—H1···O5	0.98	2.53	3.286 (5)	134
C13—H2···O4^vi^	0.98	2.59	3.496 (5)	153
C10—H5···O3^xi^	0.95	2.53	3.192 (5)	127
C8—H6···O6^x^	0.95	2.54	3.376 (5)	147
C4—H9···O2^ix^	0.95	2.48	3.401 (6)	163
C14—H10···O1	0.98	2.58	3.555 (11)	177
C27—H13···O5	0.98	2.35	3.324 (5)	172
C24—H17···O6^xii^	0.95	2.42	3.158 (5)	135
C19—H20···O4^ix^	0.95	2.59	3.482 (6)	156
C28—H22···O4	0.98	2.40	3.346 (9)	161
C28—H24···O1	0.98	2.55	3.104 (4)	116
C14—H11···Cg4	0.98	2.77	3.415 (4)	124
C27—H14···Cg1	0.98	2.69	3.419 (4)	131

Symmetry codes: (vi) *x*, *y*−1, *z*; (xi) *x*+1/2, −*y*+5/2, *z*; (x) −*x*+2, −*y*+3, *z*+1/2; (ix) −*x*+3/2, *y*+1/2, *z*+1/2; (xii) −*x*+2, −*y*+4, *z*+1/2.

## References

[bb1] Ding, C.-F., Zhang, M.-L., Li, X.-M. & Zhang, S.-S. (2006). *Acta Cryst.* E**62**, m2540–m2542.

[bb2] Flack, H. D. (1983). *Acta Cryst.* A**39**, 876–881.

[bb3] Harvey, M. A., Baggio, S., Ibañez, A. & Baggio, R. (2004). *Acta Cryst.* C**60**, m382–m385. 10.1107/S0108270104014131 15295168

[bb4] Kaes, C., Katz, A. & Hosseini, M. W. (2000). *Chem. Rev.* **100**, 3553–3590. 10.1021/cr990376z 11749322

[bb5] Oxford Diffraction (2008). *CrysAlis CCD* and *CrysAlis RED* Oxford Diffraction Ltd, Yarnton, Oxfordshire, England.

[bb6] Sheldrick, G. M. (2008). *Acta Cryst.* A**64**, 112–122. 10.1107/S0108767307043930 18156677

[bb7] Spek, A. L. (2009). *Acta Cryst.* D**65**, 148–155. 10.1107/S090744490804362X PMC2631630 19171970

[bb8] Xuan, X.-P. & Zhao, P.-Z. (2007). *Acta Cryst.* E**63**, m3180–m3181.

[bb9] Zhao, P.-Z. & Xuan, X.-P. (2007). *Acta Cryst.* E**63**, m3179.

